# Positional and dimensional osseous characteristics of the temporomandibular joint in female patients with skeletal class III malocclusion and disc displacement, with and without reduction

**DOI:** 10.3389/froh.2025.1572305

**Published:** 2025-05-15

**Authors:** Abeer A. Almashraqi, Amira A. Aboalnaga, Maged S. Alhammadi, Mona M. Salah Fayed

**Affiliations:** ^1^ Department of Clinical Oral Health Sciences, College of Dental Medicine, QU Health, Qatar University, Doha, Qatar; ^2^ Department of Orthodontics and Dentofacial Orthopedics, Faculty of Dentistry, Cairo University, Cairo, Egypt; ^3^ Orthodontics and Dentofacial Orthopedics, Department of Preventive Dental Sciences, College of Dentistry, Jazan University, Jazan, Saudi Arabia; ^4^ Department of Orthodontics, Faculty of Dentistry, Cairo University, Cairo, Egypt

**Keywords:** disc displacement, temporomandibular joints, cone-beam computed tomography, mandibular condyle, joint spaces

## Abstract

**Introduction:**

This study aimed to compare the dimensional and positional osseous features of the temporomandibular joint (TMJ) in patients with skeletal Class III malocclusion, female patients without temporomandibular disorders (TMDs), and those with TMDs presenting as disc displacement with/without reduction (DDR/DDWR).

**Methods:**

Adult patients with skeletal Class III malocclusion and average vertical facial pattern (Mandibular plane inclination = 37 ± 5°) were categorized into the TMD group comprising patients with DDR/DDWR and the non-TMD group. Three-dimensional standardized TMJ analysis was performed using cone-beam computed tomography, which included assessments of the mandibular fossa (MF), mandibular condyle, TMJ spaces, and vertical and anteroposterior intra-joint condylar positions.

**Results:**

The MF location in the DDR/DDWR group was significantly more superior (*p* < 0.0001) and anterior (*p* = 0.012) relative to the respective planes. The MF width was significantly lesser (*p* = 0.001) with a steeper (*p* < 0.0001) anterior wall inclination in the DDR/DDWR group than in the non-TMD. The mandibular condyles were located significantly more laterally (*p* = 0.016), at a greater distance from the midsagittal plane, in the DDR/DDWR group than in the non-TMD. The anterior and medial joint spaces were significantly larger (*p* < 0.0001) and the intra-joint condylar positions were more posterior and superior (*p* < 0.0001) in the DDR/DDWR group than in the non-TMD.

**Conclusions:**

The positional and dimensional osseous characteristics of the TMJ differed significantly between patients with skeletal Class III malocclusion without TMDs and those with DDR or DDWR.

## Introduction

Temporomandibular disorders (TMDs) are the most prevalent category of non-dental chronic pain conditions affecting the orofacial region (1). TMD patients present various signs and symptoms, such as pain in the temporomandibular joint (TMJ) or jaw muscles, pain during mandibular movements, TMJ sounds, locking or luxation of the TMJ, and restricted mandibular movements (2). The etiology and pathophysiology of TMDs are poorly understood since the phenomenon is multifactorial. Usually, great physiologic and external forces are absorbed within the masticatory system with no consequences; however, if these forces exceed an individual's genetic and physiological tolerance, detrimental changes may occur (2).

The prevalence of the signs and symptoms of TMDs seems to be high; the overall prevalence of clinical signs of intra-articular joint disorders was 16% in non-patient populations (3). Approximately 40–75% of them present at least one sign, and approximately 33% have at least one symptom of TMDs (4). TMDs can be broadly divided into the following two categories based on the primary source of pain and dysfunction: masticatory muscle disorders and joint disorders (2). Differentiating between muscle and articular disorders can be challenging, as muscle disorders may mimic articular issues or coexist with them. Accurate diagnosis is crucial for determining the appropriate treatment, whether surgical or non-surgical. The Diagnostic Criteria for Temporomandibular Disorders (DC/TMD) have standardized research and allowed for the classification of TMDs into the most prevalent types of muscle and joint disorders (5).

Three-dimensional (3D) imaging technology provides enhanced imaging of the TMJ structures, which aids in precise TMD diagnosis. Magnetic resonance imaging (MRI) is primarily used to evaluate the soft tissue structures of the TMJ, which is required to confirm disc displacement, disc deformity, or joint effusion disorders (6, 7). Cone-beam computed tomography (CBCT) images are also used to assess the positional and morphological osseous features of the TMJ (8–10).

Alhammadi et al. (11–13) developed a comprehensive and standardized CBCT-based 3D analysis method specifically for the TMJ. A recently published systematic review on the standardization and comprehensiveness of similar published methods concluded that this is the most standardized and comprehensive method for the 3D assessment of the TMJ (14). The analysis recorded the position, inclination, and dimensions of the mandibular fossa, condyles, and joint spaces and the intra-joint condylar positions.

Skeletal Class III malocclusion exhibits distinct mechano-dynamic characteristics when compared to skeletal Class I and II malocclusions. These include several functional deficits, such as reduced maximum bite forces, changes in occlusal contact areas, a diminished capacity to break down food, and altered mandibular kinematics (15). Additionally, individuals with skeletal Class III malocclusion display a forward disrupted positioning of the mandible; this position might negatively influence the mandibular movements and masticatory muscle activity more profoundly than Class I or II malocclusions, resulting in abnormal pressure on the TMJ (16). Subsequently, TMJ components, including the cartilage, mandibular fossa, and condyle, undergo long-lasting remodeling to adapt to the applied mechanical stress (16, 17). Thus, it is reasonable to anticipate that most TMJ configurations share similar morphological characteristics when exposed to comparable biomechanical forces within a similar craniofacial skeletal framework. Previous studies have examined the unique TMJ features in patients with and without TMDs, particularly those with Class I and II malocclusions (18). However, relevant comprehensive investigations of adult patients with TMD displaying skeletal Class III malocclusion remain scarce.

Clinically, TMDs, including DDR and DDWR, are a multifactorial disorder. However, it remains uncertain if specific morphological features of the TMJ are linked to DDR or DDWR in individuals with Class III malocclusion. The rationale of this study was to determine whether TMJ osseous morphology including its positions and dimensions should be considered a factor in the multifactorial nature of TMDs, and to pave the way for further investigations and future research in this area.

Therefore, this study was designed to compare the dimensional and positional osseous features of the TMJ in patients with skeletal Class III malocclusion and comparable vertical and transverse measurements between those patients with TMDs in the form of disc displacement with or without reduction (DDR/DDWR) and those without TMDs. The null hypothesis is that in patients with skeletal Class III malocclusion and comparable vertical and transverse measurements, the osseous TMJ positional and dimensional characteristics do not differ between those patients with TMDs in the form of DDR/DDWR and those without TMDs.

## Materials and methods

### Study design

This cross-sectional observational study was approved by the Research Ethics Committee of Cairo University in Egypt (No. 2152012). The procedures were performed according to the relevant laws and regulations. Written informed consent was obtained from each patient after the goals and methods of the study were clarified.

### Sample size and selection

The required sample size was calculated based on the reported medial joint space of the skeletal Class III patients (2.18 ± 0.72 mm) (12). The effect size was assumed to be equivalent to that of the skeletal Class II patients, wherein the medial joint space of the TMD group and non-TMD group was 3.81 ± 0.97 and 3.73 ± 0.98 mm, respectively (18). To ensure an *α*-value of 0.05 (significance level of 95%) and power of 90% (beta error of 0.2), the minimum sample size was calculated as 19 patients (38 joints) per group using G*Power 3.0.10 (University of Düsseldorf, Düsseldorf, Germany).

The inclusion criteria were (1) female patients aged between 18 and 30 years; (2) orthodontic treatment indicated with full permanent dentition present (except for the third molars); (3) skeletal Class III malocclusion (ANB ≤1°); (4) average vertical facial dimension as assessed by mandibular plane inclination, the angle formed between the sella-nasion and the gonion-menton planes (normal MP/SN = 37 ± 5°); and (5) no significant differences in the transverse skeletal maxillomandibular basal width (Norm = 16.6 ± 3 mm), as evaluated by measuring the difference between the maxillary width (distance between the right and left jugale) and mandibular effective width (distance between the right and left antegonion) (17).

Patients were excluded if they had growth abnormalities or any condylar degenerative disorders, such as hypoplasia or hyperplasia, flattening of the articular surface, subcortical sclerosis or cysts, surface erosion, osteophytes, generalized sclerosis, osteoarthritis, osteoarthrosis, and polyarthritis, which were verified on CBCT. Moreover, patients were excluded if they had transverse skeletal discrepancy or asymmetry or a history of TMJ trauma, tumors, surgery, orthodontic therapy, and orthognathic surgery.

Selected patients were divided into two groups. The TMD individuals group comprised patients with confirmed TMD in the form of DDR/DDWR, and the non-TMD group comprised patients with no history or clinical diagnosis of any TMD, such as jaw muscle myalgia, TMJ arthralgia, joint sounds, or limited mandibular movements (non-TMD group). Out of the screened one hundred and seventy two skeletal Class III malocclusion patients, 45 female patients fulfilled the selection criteria at the outpatient clinic of the Orthodontic Department, Faculty of Dentistry, Cairo University, Egypt.

### Clinical examination

Two examiners conducted clinical examinations under the direct supervision of an experienced TMD specialist. Before commencing the research, measurements of a pilot sample (20 patients) were recorded by the two examiners and calibrated with the specialist's measurements to calculate the inter-observer reliability. An accurate diagnosis was performed for every patient using a customized history and examination chart following the original English version of the DC/TMD (5). Clinical evaluations of the enrolled patients included (1) TMJ palpation, (2) masticatory muscle palpation, (3) mandibular movement evaluation, and (4) TMJ sound assessment. Based on the clinical evaluation, Group 1 comprised 21 patients with DDR/DDWR (42 TMJs), while Group 2 comprised 24 patients without TMDs (48 TMJs). The TMD group presented (1) DDR characterized by an absence of joint pain, reproducible opening, or closing clicks with reciprocal clicking as a key criterion or (2) DDWR characterized by a history of locking or catching that interfered with eating, an absence of TMJ clicking, unassisted mouth opening (even painful) ≤35 mm, passive stretch ≤4 mm (hard-end feel), and contralateral excursion <7 mm or uncorrected ipsilateral deviation upon opening. The selected patients were considered only if both sides are affected in group 1 or both sides are unaffected in group 2 to avoid any possible asymmetry.

### CBCT analysis

Each patient underwent imaging using a next-generation i-CAT CBCT unit (Imaging Sciences International, Hatfield, US). The machine was set with the following exposure parameters: current flow, 18.54 mA at 120 kV; exposure time, 8.9 s; voxel size, 0.30 mm; slice thickness, 2 mm; and field of view, 23 × 17 cm. Patients were oriented with a natural head position using a band to position the head against the head rest and chin cup. The mid-sagittal plane was positioned perpendicular to the horizontal plane using the vertical and horizontal alignment laser beams as recommended by the manufacturer. During the scanning process, the patients were instructed to keep the mandible closed with maximum dental intercuspation and to avoid swallowing or any movement.

All participants had skeletal Class III malocclusion, most of which required either orthognathic surgery (single or combined jaws) or camouflage treatment. Thus, the CBCT images were used as pre-treatment records required for orthodontic treatment without the need for any other radiographic records. The acquired CBCT images were converted to Digital Imaging and Communications in Medicine format and imported into the image processing software (Anatomage version 5.01, Anatomage, San Jose, CA, USA). Following this, fully reconstructed 3D volumetric images were generated. All landmarks were located on 3D volumetric images, and refinement of landmark localization was performed using the generated multiplanar slice locator in the three planes of space (11–13, 18, 19) (Supplementary 1). The 3D reference planes were identified as described by Swennen et al. (20) (Supplementary 2).

**Figure 1:**
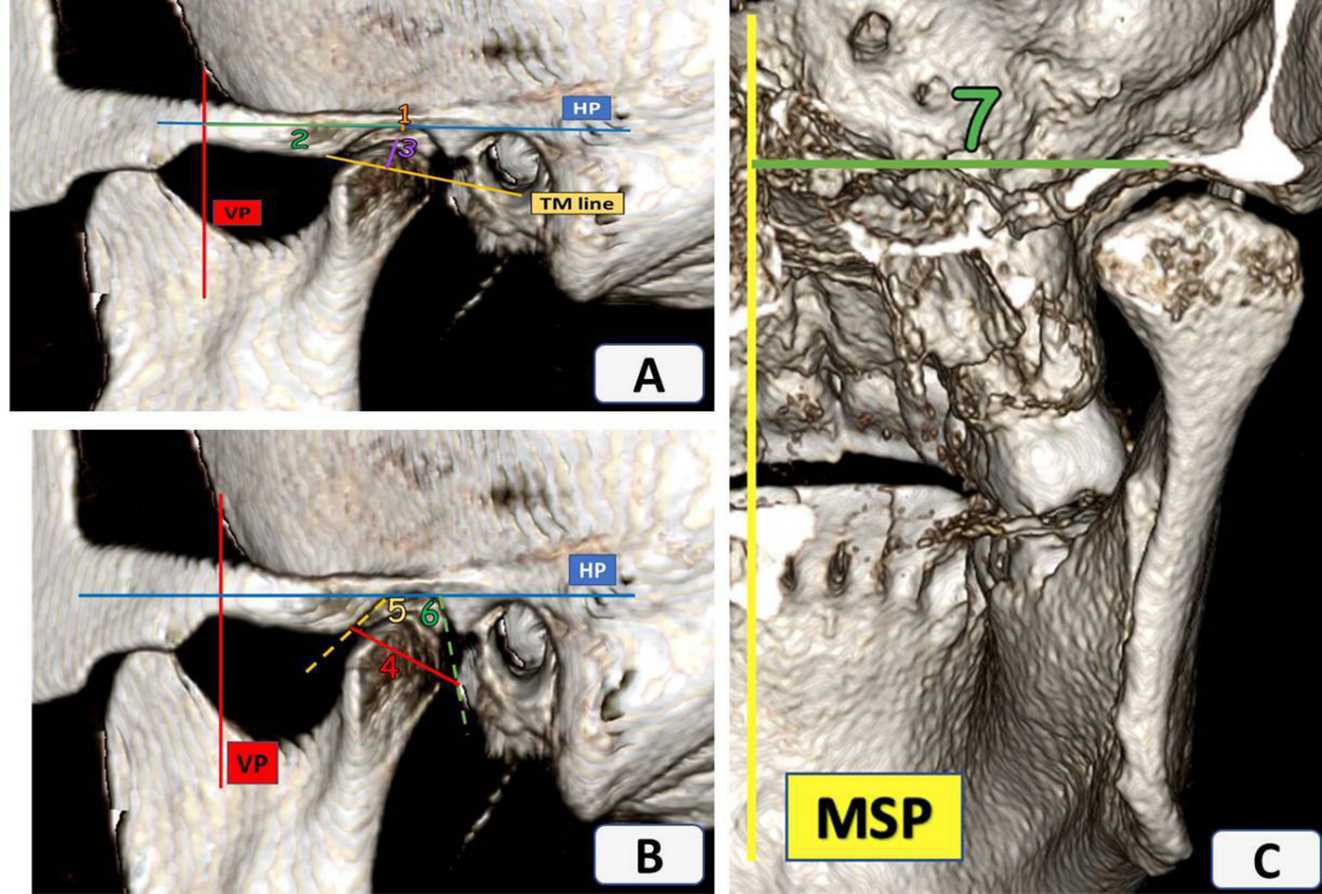
Sagittal and coronal views of the temporomandibular joint. **(A)** 1—Mandibular fossa vertical position (MFPVP), 2—Mandibular fossa anteroposterior position (MFPAP), 3—Mandibular fossa height (MFH); **(B)** 4—Mandibular fossa width (MFW), 5—Mandibular fossa anterior wall inclination (AFLHP), 6—Mandibular fossa posterior wall inclination (PFLHP); **(C)** 7—Mandibular fossa mediolateral position (MFPML).

**Figure 2:**
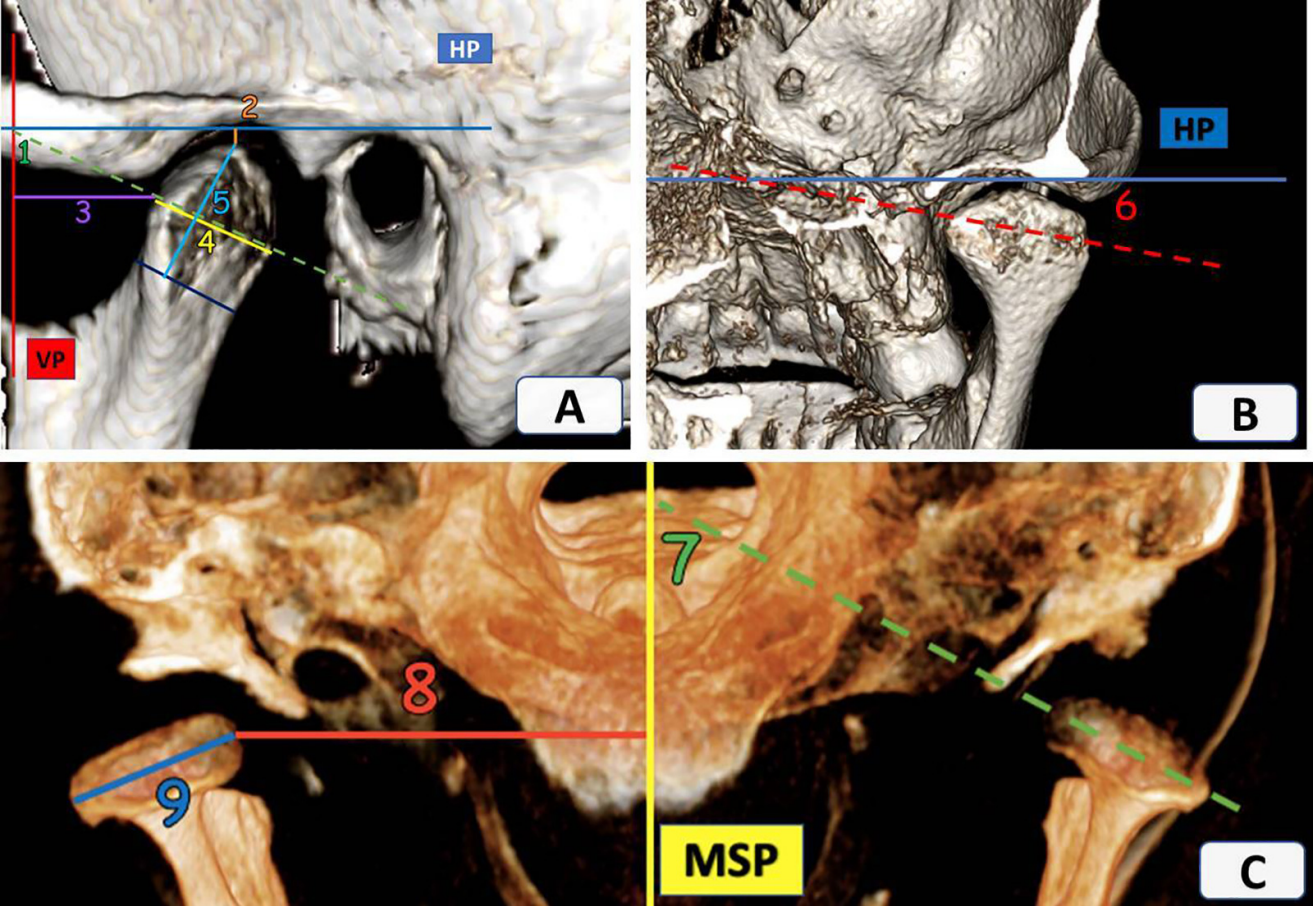
Sagittal, coronal, and axial views of the temporomandibular joint. **(A)** 1—Mandibular condyle vertical inclination (VCI), 2—Mandibular condyle vertical position (VCP), 3—Mandibular condyle anteroposterior position (APCP), 4—Condylar width (CW), 5—Condylar height (CH); **(B)** 6—Mandibular condyle horizontal inclination (HCI); **(C)** 7—Mandibular condyle anteroposterior inclination (APCI), 8—Mandibular condyle mediolateral position (MLCP) and 9—Condylar length (CL).

**Figure 3:**
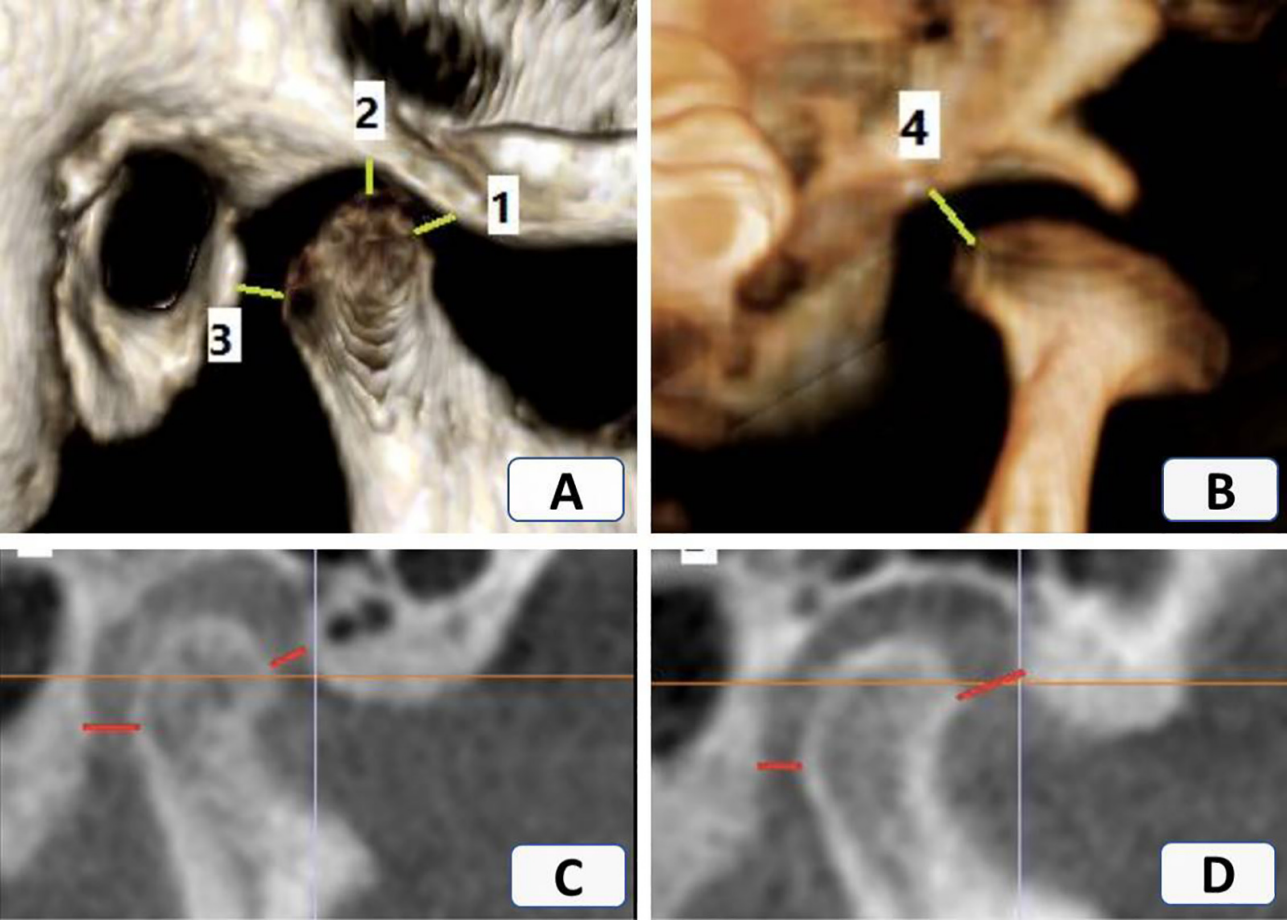
Sagittal and coronal views of the temporomandibular joint. **(A)** 1—Anterior joint space (AJS), 2—Superior joint space (SJS), 3—Posterior joint space (PJS); **(B)** 4—Medial joint space (MJS); **(C)** Anterior intra—joint condylar position (positive value); **(D)** Posterior intra—joint condylar position (negative value).

A standardized TMJ analysis was adopted for this study, as described by Alhammadi et al. (11–13, 18). All linear and angular measurements were conducted in the 3D volumetric images by oral and maxillofacial radiologist and an orthodontist (Supplementary 2). The analysis included mandibular fossa measurements (Figure 1), mandibular condyle measurements (Figure 2), TMJ spaces (Figure 3), and the vertical and anteroposterior intra-joint condylar positions, which were calculated based on the formula developed by Pullinger and Hollender (21). To assess the significance of any errors during measurement, 10% of the patients were measured by the same operator twice at an interval of 2 weeks and once by another operator to calculate the intra- and inter-observer reliabilities. Although blinding the clinical examinations was not possible, all CBCT measurements were performed without revealing the patients’ identities during the CBCT analysis.

### Statistical analysis

Statistical analyses were performed using IBM Statistical Product and Service Solutions software, version 26.0, for Windows (IBM Corp., Armonk, NY, USA). Intra-class correlation (ICC) was used to assess the intra- and inter-observer reliabilities. Data were assessed for normality using the Shapiro–Wilk test and were considered normally distributed if the *P*-value was >0.05. An independent *t*-test was used to compare normally distributed quantitative data between the groups. Statistically data were handled for the patients not the joints by averaging both sides, this is because both sides of DDR/DDWR group were affected and the same for the non-TMD group, both sides are free from TMD signs and symptoms. *P*-values <0.05 were considered statistically significant.

## Results

Out of the whole examined non-consecutive sample, only 21 female patients were considered in group 1 (DDR/DDWR), while patients without TMDs comprised of 24 female patients (Group 2) with a mean age of 22.48 ± 6.63 years. All measurements showed excellent intra- and inter-observer reliabilities (ICC > 0.75). No significant differences in the baseline anteroposterior, vertical and transverse measurements were detected between the two groups. The DDR/DDWR and non-TMD groups had mean ANB angles of −1.07 ± 1.29 and −1.72 ± 1.26° (*P* = 0.098), mean mandibular plane angles of 33.77 ± 4.95 and 35.19 ± 6.49° (*P* = 0.41), and mean maxillomandibular transverse discrepancies of 16.52 ± 1.24 and 15.41 ± 2.41 mm (*P* = 0.249), respectively.

The mandibular fossa position and parameters differed significantly between the groups (Table 1). Compared to the non-TMD group, the location of the mandibular fossa patients with DDR/DDWR was significantly more superior (*P* < 0.0001) and anterior (*P* = 0.012) relative to the respective planes. Furthermore, the mandibular fossa width in patients with DDR/DDWR was significantly lesser than in the non-TMD group (17.34 ± 1.67 vs. 19.2 ± 1.99 mm; *P* = 0.001). Patients with DDR/DDWR also showed a significantly steeper anterior wall inclination and flatter posterior wall inclination of the mandibular fossa than the non-TMD group (*P* < 0.0001 and *P* = *0*.04, respectively).

**Table 1:** Comparative statistical analysis of the mandibular fossa measurements between TMD and Non-TMD skeletal class III groups.

Group		DDR/DDWR		Non-TMD		*P*-Value
Mandibular fossa measurements		Mean	SD	Mean	SD	
Mandibular fossa position (mm)	MFPVP	1.76	0.89	3.01	1.22	<0.0001**
	MFPAP	9.08	2.96	11.41	2.99	0.012*
	MFPML	47.16	2.79	46.70	1.14	0.485
Mandibular fossa parameters	MFH (mm)	8.74	1.41	8.52	0.96	0.544
	MFW (mm)	17.34	1.67	19.20	1.99	0.001**
	AFLHP (^o^)	51.49	12.09	38.38	6.09	<0.0001**
	PFLHP (^o^)	51.18	8.79	57.71	11.43	0.040*

* Significant (*P* < .05).

** Highly significant (*P* < .001).

The mandibular condylar measurements of the groups are shown in Table 2. The condylar inclination values varied significantly between the two groups. Patients with DDR/DDWR showed significantly increased horizontal and vertical inclinations (*P* = 0.047 and *P* = 0.002, respectively) and significantly decreased anteroposterior condylar inclination (*P* < 0.0001) compared to the non-TMD group. As for the condylar position, the vertical and anteroposterior condylar positions did not vary significantly between the groups. However, the mediolateral condylar position differed significantly (*P* = 0.016), and the medial pole of the condyle in the DDR/DDWR group was located laterally at a significantly greater distance from the midsagittal plane than in the non-TMD group (40.85 ± 3.15 vs. 38.9 ± 1.96 mm).

**Table 2:** Comparative statistical analysis of the mandibular condyle measurements between TMD and Non-TMD skeletal class III groups.

Group		DDR/DDWR		Non-TMD		*P*-Value
Mandibular condyle measurement		Mean	SD	Mean	SD	
Mandibular condyle inclination (^o^)	HCI	6.36	3.32	4.54	2.64	0.047*
	VCI	79.09	6.90	71.31	8.95	0.002*
	APCI	73.36	4.52	82.16	4.55	<0.0001**
Mandibular condyle position (mm)	VCP	1.66	1.18	2.00	0.97	0.310
	APCP	4.52	2.21	5.90	2.69	0.071
	MLCP	40.85	3.15	38.90	1.96	0.016*
Mandibular condyle parameters (mm)	CL	19.22	2.70	17.38	1.72	0.009*
	CW	6.81	1.12	7.45	1.45	0.106
	CH	9.56	1.79	10.81	0.79	0.007*

* Significant (*P* < .05).

** Highly significant (*P* < .001).

In patients with DDR/DDWR, the condylar length was significantly greater (*P* = 0.009) and the condylar height was significantly smaller in the DDR/DDWR group compared to the other non-TMD group (*P* = 0.007). The condylar width did not differ significantly between the groups. Regarding the joint spaces and intra-joint condylar positions, the anterior and medial joint spaces were significantly greater in the DDR/DDWR group than in the non-TMD group (*P* = 0.000). The superior and posterior joint spaces were significantly greater and smaller in the patients with DDR/DDWR than in the non-TMD group (*P* = 0.002 and *P* = 0.01, respectively). For the intra-joint condylar positions, patients with DDR/DDWR showed more posterior and superior compared to the non-TMD group (*P* < 0.0001) (Table 3).

**Table 3:** Comparative statistical analysis of the temporomandibular joint spaces and intra-joint condylar position measurements between TMD and Non-TMD skeletal class III groups.

Group	DDR/DDWR		Non-TMD		*P*-Value
Mandibular joint spaces and intra-joint condylar position measurements (mm)	Mean	SD	Mean (mm)	SD	
AJS	3.02	0.82	1.97	0.42	<0.0001**
SJS	3.69	0.97	2.92	0.33	0.002**
PJS	2.29	0.72	2.86	0.70	0.011*
MJS	4.21	1.31	2.17	0.48	<0.0001**
APJCP	−13.27	22.19	15.43	21.39	<0.0001**
VJCP	2.40	0.46	3.69	0.97	<0.0001**

* Significant (*P* < .05).

** Highly significant (*P* < .001).

## Discussion

The sample for this study was limited to female patients, as females were selected due to the higher prevalence of TMDs in this group compared to males (22). Previous studies have examined the osseous characteristics of the TMJ in non-TMD individuals with various anteroposterior and vertical skeletal combinations. These studies deduced that each skeletal pattern uniquely remodels the TMJ as an adaptive response to the acting mechanical stress, leading to characteristic positional and morphological differences in the TMJ (23–25). According to Alhammadi et al. (12), individuals with skeletal Class III malocclusion have the highest mandibular fossa width, most inferiorly positioned fossa, and lowest tubercular height when compared to those patients with Class I and II malocclusions. According to Arieta-Miranda et al. (23) and Katsavrias and Halazonetis (26), the condyles were closer to the roof of the glenoid fossa in Class III than in Class I and II patients.

Many studies have also shown that the smallest joint spaces occur in Class III malocclusion patients (12, 23, 27, 28), which may be secondary to the condylar surface area being the largest (12). Considering these findings, this study evaluated the TMJ structures in adult patients with skeletal Class III malocclusion with similar transverse skeletal and vertical malocclusions, and DDR/DDWR group compared to the other non-TMD group. Hyperdivergent and hypodivergent patients were excluded because both the condylar position and morphology vary according to the vertical facial morphology (24, 25).

A 3D TMJ analysis was employed (11–13), which is more comprehensive and standardized compared to other published methods (14). In addition to a precise description of the TMJ landmarks for accurate localization, the analysis involved stable and reproducible craniofacial reference lines that were defined by distant and mostly unchangeable cranial base landmarks (horizontal, midsagittal, and vertical planes) (20). A previous TMJ analysis by Vitral et al. (29) used a tuberculo-meatal reference line, which was determined by two anatomical landmarks. However, the articular tubercle was confirmed to be affected by growth and aging (30, 31), while another study showed that the inferior meatus was shown to vary in localization between successive sagittal slices (32).

This study revealed significant differences between the TMJs with and without DDR/DDWR. Regarding the mandibular fossa position, the DDR/DDWR group showed more superior and anterior positions of the fossae relative to their respective planes compared to the non-TMD group. This could be attributed to the presence of an anteriorly displaced disc that results in the condyles exerting more force against the upper and anterior walls of the mandibular fossa, inducing bone remodeling in the corresponding direction (33, 34). Moreover, the mandibular fossa showed a lesser width and a steeper articular tubercle in the DDR/DDWR group than in the non-TMD group. These anatomical variations in the TMJ structures are consistent with internal derangement disorders (31, 35). The increased steepness of the articular eminence in the DDR/DDWR group might be due to the disc displacement followed by remodeling of the glenoid fossa or the normal bone remodeling process; however, a longitudinal study is warranted to confirm this (33).

Significant differences in the condylar morphology were detected between the groups. These differences could have resulted from the morphology of the condylar inclines affecting the position of the disc itself or the long-term repositioning of the disc affecting the condylar inclines. Furthermore, the condyle was more laterally positioned in the DDR/DDWR group than in the non-TMD group, which was determined by larger medial joint spaces and might indirectly indicate that the disc displacement occurs in the anterior and medial directions. This could also explain the reduced condylar height observed in the DDR/DDWR group. Pathological friction between the condylar head and roof of the fossa might have caused some condylar resorption.

Alhammadi et al. (33) reported more vertical condylar inclination and greater medial joint spaces in patients with Class II malocclusion and TMD compared to the non-TMD group. Similarly, Talaat et al. (36) found a positive correlation between TMD and the flattening of the condylar head. Interestingly, the glenoid fossa roof was thicker in patients with TMD than in those patients without, which compensated for the increased mechanical stress on the TMJ (37, 38).

Significant intergroup differences were found in the joint spaces and intra-joint condylar positions. Due to articular disc displacement, the condyle assumes a posterior, superior, and lateral intrajoint position. Many CBCT-based studies (18, 33, 39, 40) have found that the condyles in individuals with TMD are positioned non-centrically in the glenoid fossa. These significant differences were also reflected in the corresponding joint spaces, where the anterior and medial joint spaces were significantly increased in the affected joints. Ikeda and Kawamura (41) indicated that alteration of the disc position, particularly the posterior band position, leads to changes in the joint spaces in CBCT images.

Other TMD disorders might be presented with similar or different features compared to the non-TMD group. Campos et al. investigated temporomandibular joint (TMJ) pain and the magnetic resonance imaging (MRI) characteristics in a sample of 104 TMJs with degenerative changes and 58 TMJs without such changes. The degenerative changes examined include osteophytes, erosion, avascular necrosis, subcondral cysts, and intra-articular loose bodies. They reported that Flattening, retropositioning, and hypomobility of the condyle showed no significant differences concerning the presence or absence of degenerative bony changes. However, retropositioning of the condyle was significantly associated with disk displacement with reduction, while condylar hypomobility was significantly more common in TMJs with disk displacement without reduction (42).

The null hypothesis was rejected; it is worth mentioning that there were significant differences in most of the components of the TMJ, especially the condylar and the joint spaces between Class III patients with DDR/DDWR and those without TMD. This should be thoroughly investigated in the future using more advanced imaging modalities.

There are several limitations to be mentioned; although being calculated in advance, a greater sample size that includes both genders is recommended. The mandibular growth might continue to the mid-twenties, so a higher age group might make a difference. The diagnostic information obtained was limited to specific osseous TMJ components. To assess soft-tissue positions and abnormalities, MRI is recommended for a thorough examination of the differences in soft tissues between the groups. This approach allows for the recruitment of more patients in each subgroup, facilitating valid comparisons between DDR and DDWR. Another limitation is the cross-sectional study design; a long-term follow-up study may yield different findings, particularly concerning the remodeling of the TMJ structures. The impact of various confounding variables (such as gender, age, psychosocial factors, para-functional activities, etc.) needs to be examined to assess their influence on temporomandibular disorders (TMD) or to confirm their relationship with TMD.

## Conclusions

Within its limitations, this study showed that the positional and dimensional characteristics of the TMJ differed significantly between Class III patients with DDR/DDWR and those without any history of signs and symptoms of TMDs or clinical diagnosis of TMDs. Compared with patients without TMD, those patients with DDR/DDWR showed more superior and anterior locations of the mandibular fossa, lesser mandibular fossa width, steeper anterior wall inclination, and flatter posterior wall inclination. The condyles were situated more laterally in the DDR/DDWR group than in the non-TMD group. Since the anterior and medial joint spaces were significantly greater in patients with DDR/DDWR than in those patients without, the intra-joint condylar positions were more posterior and superior in the former than in the latter. From a clinical perspective, TMD specialists should thoroughly evaluate the hard tissue component of TMJ morphology before proceeding with any intervention to reposition the displaced disc, this correction might be temporary due to underlying factors, including, but not limited to, TMJ morphology. All of these findings should be interpreted with caution, as TMD is multifactorial, making it impossible to eliminate all known or unknown factors. Assessing cases with TMD prior to orthodontic treatment or orthognathic surgical intervention is crucial to prevent complications that may arise from worsening disc position after these therapies. The use of a disc-specific imaging methodology like MRI might provide a better insight into the required precaution before any dental or TMJ intervention. Morphological and quantitative analysis is needed on a larger sample, which would also allow the analysis of the eventual differences between the variations in the displacement of the DDR and DDWR discs.

## Data Availability

The data analyzed in this study is subject to the following licenses/restrictions: based on the request from corresponding author. Requests to access these datasets should be directed to abeer.almashraqi@qu.edu.qa.
