# Supplementary material for: Positional and dimensional osseous characteristics of the temporomandibular joint in female patients with skeletal class III malocclusion and disc displacement, with and without reduction
Source: Front Oral Health. 2025 May 15;6:1572305. doi: 10.3389/froh.2025.1572305 (PMC12119597; doi:10.3389/froh.2025.1572305)
Supplement: Supplementary file 1 [file Table1.docx]

**Supplementary 1:** Three-dimensional temporomandibular joint landmarks used in the study

| **Landmark** | **Definition** |
| --- | --- |
| **MF** | The most superior and midpoint of the hard tissue mandibular fossa region. |
| **AT** | The most inferior point of articular tubercle. |
| **IM** | The most inferior point of internal auditory meatus. |
| **AFPi** | The most anterior and inferior point in the anterior wall of the mandibular fossa. |
| **AFPs** | The most superior point in the anterior wall of the mandibular fossa. |
| **PFPi** | The most posterior and inferior point in the posterior wall of the mandibular fossa. |
| **PFPs** | The most superior point in the posterior wall of the mandibular fossa. |
| **SCP** | The most superior point of the condylar head. |
| **LCP** | The most lateral point of the condylar head. |
| **MCP** | The most medial point of the condylar head. |
| **ACP** | The most anterior point of the condylar head. |
| **PCP** | The most posterior point of the condylar head. |
| **MJSF** | The most lateral point of the medial wall of mandibular fossa. |
| **AJSF** | The most posterior point of the anterior wall of the mandibular fossa opposing the shortest anterior condylar-fossa distance. |
| **AJSC** | The most anterior point of the condyle opposing the shortest anterior condylar-fossa distance. |
| **PJSF** | The most anterior point of the posterior wall of the mandibular fossa opposing the shortest posterior condylar-fossa distance. |
| **PJSC** | The most posterior point of the condyle opposing the shortest posterior condylar-fossa distance. |
