# Supplementary material for: Positional and dimensional osseous characteristics of the temporomandibular joint in female patients with skeletal class III malocclusion and disc displacement, with and without reduction
Source: Front Oral Health. 2025 May 15;6:1572305. doi: 10.3389/froh.2025.1572305 (PMC12119597; doi:10.3389/froh.2025.1572305)
Supplement: Supplementary file 2 [file Table2.docx]

**Supplementary 2:** Reference planes and temporomandibular joint measurements used in the study

| **Reference Planes** | | |
| --- | --- | --- |
| **HP** | Horizontal plane | Plane joining 3 landmarks; right orbitale, right and left porions. |
| **MSP** | Midsagittal Plane | Plane passing through sella and nasion, constructed perpendicular to the horizontal plane. |
| **VP** | Vertical Plane | Plane passing through sella and perpendicular to the sagittal and horizontal planes. |
| **TM** | Tuberculo-metal Line | Line between AT and IM. |
| **Temporomandibular Joint Measurements** | | |
| **Mandibular fossa measurements** | | |
| **MFVP** | Mandibular fossa vertical position | The perpendicular distance between MF and HP plane. |
| **MFAP** | Mandibular fossa anteroposterior position | The perpendicular distance between MF and VP plane. |
| **MFML** | Mandibular fossa mediolateral position | The perpendicular distance between MF and MSP plane. |
| **MFH** | Mandibular fossa height | The perpendicular distance between MF and TM line. |
| **MFW** | Mandibular fossa width | The horizontal distance between AFPi and PFPi. |
| **AFLHP** | Mandibular fossa anterior wall inclination | The angle between AFPi - AFPs line and HP plane. |
| **PFLHP** | Mandibular fossa posterior wall inclination | The angle between PFPi - PFPs and HP plane. |
| **Mandibular condyle measurements** | | |
| **HCI** | Horizontal condylar inclination | Angle between MCP - LCP line and HP plane. |
| **VCI** | Vertical condylar inclination | Angle between ACP-PCP line and VP plane. |
| **APCI** | Anteroposterior condylar inclination | Angle between MCP-LCP line and MSP plane. |
| **VCP** | Vertical condylar position | Perpendicular distance between SCP and HP plane. |
| **APCP** | Anteroposterior condylar position | Perpendicular distance between ACP and VP plane. |
| **MLCP** | Mediolateral condylar position | Perpendicular distance between MCP and MSP plane. |
| **CL** | Condylar length | The distance between MCP and LCP. |
| **CW** | Condylar width | The distance between ACP and PCP. |
| **CH** | Condylar height | The perpendicular distance between SCP and the line joining the constricted condylar neck points. |
| **TMJ space measurements** | | |
| **AJS** | Anterior Joint Space | The distance between AJSC-AJSF. |
| **PJS** | Posterior joint space | The distance between PJSC-PJSF. |
| **SJS** | Superior joint space | The distance between SCP-MF. |
| **MJS** | Medial joint space | The distance between MCP-MJSF. |
| **VCJP** | Vertical condylar intra-joint position | The difference between SCP to TM line and MF to TM line. |
| **APCJP** | Anteroposterior condylar intra-joint position | Anteroposterior position of condyle within the mandibular fossa as determined by Pullinger and Hollander equation ^18^, which is the difference between PJS and AJS. |
